# Neural sensitivity to peer rejection predicts depressive symptoms and daily mood in adolescents

**DOI:** 10.1038/s44277-026-00073-0

**Published:** 2026-10-22

**Authors:** Nayoung Kim, Isaac N. Treves, Lilian Y. Li, Sarah E. Sarkas, Katherine Durham, David Pagliaccio, Nicholas B. Allen, Stewart A. Shankman, Randy P. Auerbach

**Affiliations:** 1 Department of Psychiatry, Columbia University, New York, NY US; 2 Department of Psychiatry, The Ohio State University, Columbus, OH, US; 3 Department of Psychiatry, Northwestern University, Chicago, IN US; 4 Department of Psychology, University of Oregon, Eugene, OR US

**Keywords:** Predictive markers, Social behaviour

## Abstract

Understanding the role of peer feedback, particularly rejection, is critical for adolescent major depressive disorder (MDD). However, the neurophysiological markers core to peer feedback, especially those that may presage risk for depression, are unclear. Adolescents (*N* = 179; ages 13–18; healthy controls *n* = 62, remitted MDD *n* = 80, current MDD *n* = 37) completed a baseline clinical assessment and a revised Chatroom Task during EEG recording, from which the P3 to peer acceptance and rejection feedback was quantified. Over 12 months, daily mood and keyboard inputs from adolescents’ smartphones were passively obtained. Linear models evaluated whether the P3 difference between rejection and acceptance (ΔP3) prospectively predicted depressive severity and average daily mood. Exploratory natural language processing examined whether friendship-focused social communication moderated the association between neural sensitivity to peer feedback and subsequent clinical outcomes. Group and feedback type interacted in predicting P3 amplitude (F(2176) = 4.84, *p* = 0.009): adolescents with current MDD showed larger P3 amplitudes to rejection than acceptance, whereas remitted MDD showed elevated amplitudes to both feedback types relative to controls. In a general linear model adjusting for age, sex, site, and baseline depression severity, the Grou*p* × ΔP3 interaction was significant in predicting severity at follow-up (b = 0.092, *p* = 0.045); higher ΔP3 predicted higher follow-up severity in current MDD, but not in remitted MDD or healthy controls. Across the full sample, larger ΔP3 was associated with lower average daily mood over 12 months (b = −1.673, *p* = 0.045). These findings suggest that enhanced neural sensitivity to peer rejection may be deeply engrained in adolescent depression, shaping both the longitudinal course of depressive symptoms and daily mood.

## Lay Summary

Being rejected by peers is especially painful for teens with depression. We measured brain responses to peer acceptance and rejection in 179 adolescents, then tracked their mood through their smartphones for a year. Teens with current depression showed stronger brain responses to rejection than acceptance. Larger rejection responses predicted worsening depressive symptoms in these teens, and lower daily mood across all participants. Heightened brain sensitivity to rejection may identify adolescents at risk and guide treatment.

## Introduction

Major depressive disorder (MDD) is one of the most prevalent psychiatric conditions in adolescence [1]. During this developmental period, social orientation shifts toward peers, with friendships and broader peer networks becoming increasingly central to identity, belonging, and social standing [2, 3]. This reorganization of social priorities unfolds in parallel with a marked increase in depression risk [1, 4, 5]. Adolescents vary substantially in how strongly they respond to peer evaluation, including experiences of acceptance and rejection, and this variability is thought to contribute to differential risk for depressive outcomes [4, 6, 7]. Among adolescents with, or at elevated risk for, MDD, sensitivity to negative social cues appears especially pronounced and clinically consequential [4, 7, 8]. Heightened responsiveness to perceived rejection may amplify low mood, disrupt peer relationships, and promote persistent patterns of social disconnection; processes that have been implicated in both the onset and chronic course of depression in high-risk youth [9, 10]. Psychological and neural sensitivities to social rejection have, therefore, been proposed as key vulnerabilities that amplify and maintain depressive symptoms in the context of negative peer experiences [6, 7]. However, few studies have prospectively tested whether neural responses to peer feedback, particularly rejection, predict the longitudinal course of adolescent depression [4, 11, 12].

Electroencephalography (EEG) provides a temporally precise index of social-evaluative processing, resolving peer feedback responses at the millisecond level [13, 14]. Among feedback-locked ERPs, we focus on the P3, a centroparietal positivity peaking approximately 300–600 ms after feedback onset, because it indexes the motivational salience and self-relevance of an outcome [14, 15]. Peer-feedback paradigms reliably elicit a centroparietal P3 to evaluative outcomes; however, the direction of the acceptance–rejection difference has been inconsistent across studies. A minority of studies report a larger P3 to rejection,[16, 17] others report a larger P3 to acceptance [18, 19], and the most common finding is no reliable valence difference [15, 20]. Although valence effects on the feedback P3 are heterogeneous across paradigms, the studies most comparable to the present design, personalized chatroom-style tasks delivering explicit acceptance and rejection feedback from ostensibly real peers, link an enhanced rejection-related P3 to individual differences in social-evaluative sensitivity [16, 17]. This pattern is consistent with the P3 indexing the motivational salience of outcomes, as rejection is the more self-relevant and threatening cue for adolescents with or at elevated risk for depression [16, 17]. Most prior work, however, has been cross-sectional and limited to comparisons of current MDD with healthy controls. It remains unclear whether enhanced rejection-related differentiation is also present in remitted MDD, which would indicate a trait-like vulnerability, and whether the differential P3 response to rejection relative to acceptance prospectively predicts the course of everyday mood and depressive symptoms. Addressing these gaps requires moving beyond single, clinic-based assessments to intensive, ecologically valid measurement of adolescents’ mood and social interaction in daily life.

Advances in digital phenotyping now make it possible to characterize adolescents’ affective experience and social behavior in daily life with much greater precision [21–24]. Smartphone-based experience sampling methods (ESM) can capture within-person fluctuations in mood across days and weeks, and repeated ratings of daily affect have been linked to both current symptoms and future risk [24–27]. Additionally, text- and keyboard-based measures from smartphones can also be analyzed using tools such as linguistic dictionaries and topic modeling to quantify naturalistic language and peer-related themes, and these linguistic features show robust associations with depression and other mental health outcomes [22, 23, 28, 29]. Beyond the neural response itself, the broader social context in which adolescents engage with peers may shape whether heightened rejection sensitivity translates into worsening symptoms. Friendship-focused communication, the proportion of social communication containing friend-related language, indexes ongoing positive peer engagement, and lower levels of such engagement have been linked to social disengagement and depressive risk [10, 30, 31]. Given that texting is adolescents’ primary mode of peer contact, smartphone-derived indices of friend-focused messaging may capture real-world social disengagement relevant to depressive outcomes [32, 33]. Within this framework, integrating neural markers of social-evaluative processing with smartphone-derived indices of mood and communication offer a powerful approach for testing how brain-level sensitivity to peer rejection translates into real-world emotional functioning and the longitudinal course of adolescent depression [22, 29, 34].

Directly addressing these gaps, this study integrated EEG-based indices with smartphone-derived measures of mood and communication to test whether adolescents’ neural sensitivity to peer-evaluative feedback is associated with their longitudinal course of depression and everyday functioning (i.e., daily mood, smartphone communication). At baseline, adolescents with current MDD, remitted MDD, and no history of depression completed a revised Chatroom task during EEG recording, from which we quantified the P3 to each feedback type [16, 20]. Depressive symptoms were assessed at baseline and the 12-month follow-up using the Children’s Depression Rating Scale–Revised (CDRS-R; [35]. Over the 12-month follow-up period, daily mood ratings and smartphone keyboard inputs characterizing patterns of friendship-focused communication were passively obtained. It was hypothesized that: (1) the P3 to rejection relative to acceptance would be most elevated among adolescents with current MDD, followed by those with remitted MDD, and then healthy controls; (2) among adolescents with a history of MDD, larger baseline ΔP3 would predict a worse course of depressive symptoms and daily mood over 12-month follow-up. In exploratory analyses, the study further examined whether the frequency of friendship-related language in smartphone social communication moderated the association between ΔP3 and later depressive severity, testing whether ongoing positive peer engagement buffers the downstream impact of heightened neural rejection sensitivity. By bridging neural and behavioral markers, this study aims to clarify how social-evaluative neural responses shape both symptom trajectories and everyday emotional and social functioning in adolescents with and without a history of MDD.

## Methods and materials

### Participants

Participants were recruited as part of an ongoing longitudinal study examining social processing deficits in adolescent depression [22–24]. Adolescents ages 13–18-years-old were enrolled from communities in New York, NY, and Chicago, IL. The inclusion criteria were: (a) Tanner Stage ≥3 [36], (b) fluency in English, (c) a Wechsler Abbreviated Scale of Intelligence-II (WASI-II) score ≥85 [37], (d) ownership of a personal smartphone (Android or iOS), and (e) right-handedness. General exclusion criteria included: (a) history of head injury, seizures, or other neurological disorders, (b) current moderate or severe substance use disorder, and (c) lifetime history of bipolar or psychotic disorders, oppositional defiant disorder, conduct disorder, organic mental disorder, or developmental disorder (e.g., autism).

Additional inclusion criteria were applied to the two clinical groups (current MDD and remitted MDD). Youth in the current MDD group met criteria for a current major depressive episode and/or persistent depressive disorder on the Kiddie Schedule for Affective Disorders and Schizophrenia–Present and Lifetime Version (K-SADS-PL) [38] and had a CDRS-R [35] score ≥ 55. Youth in the remitted MDD group had a past depressive episode (i.e., at least five symptoms for ≥ 2 weeks), but no depressive disorder in the past 2 months and a CDRS-R score ≤ 54. A total of 194 adolescents were enrolled, including current MDD (*n* = 39), remitted MDD (*n* = 88), and healthy controls (*n* = 67). Sociodemographic and clinical characteristics are summarized in Table 1.

**Table 1:** Clinical and Sociodemographic Characteristics Stratified by Group.

	Total Sample	Healthy Adolescents	Adolescents with remitted MDD	Adolescents with current MDD	*p*
	*N* = 194	*n* = 67	*n* = 88	*n* = 39	
**Age**	16.69 (1.56)	16.44 (1.49)	16.62 (1.47)	17.30 (1.06)	0.872
**Sex (% female)**	156 (80.41%)	53 (79.10%)	75 (85.23%)	28 (71.79%)	0.320
**Race**					0.057
White	92 (47.42%)	25 (37.31%)	52 (59.09%)	15 (38.46%)	
Asian	31 (15.98%)	10 (14.93%)	11 (12.50%)	10 (25.64%)	
Black/African American	24 (12.37%)	12 (17.91%)	7 (7.95%)	5 (12.82%)	
American Indian/Alaska Native	2 (1.03%)	0 (0.00%)	1 (1.14%)	1 (2.56%)	
Native Hawaiian or other Pacific Islander	2 (1.03%)	0 (0.00%)	1 (1.14%)	1 (2.56%)	
More than one race	26 (13.40%)	14 (20.90%)	9 (10.23%)	3 (7.69%)	
Unknown or Not reported	17 (8.76%)	6 (8.96%)	7 (7.95%)	4 (10.26%)	
**Family income**					0.628
Less than $25,000	13 (6.70%)	5 (7.46%)	5 (5.68%)	3 (7.69%)	
$25,000 - $50,000	26 (13.40%)	11 (16.42%)	11 (12.50%)	4 (10.26%)	
$50,000 - $75,000	21 (10.82%)	9 (13.43%)	8 (9.09%)	4 (10.26%)	
$75,000 - $100,000	21 (10.82%)	10 (14.93%)	11 (12.50%)	0 (0.0%)	
$100,000 or more	90 (46.39%)	29 (43.28%)	50 (56.82%)	11 (28.21%)	
Unknown or Not Reported	23 (11.86%)	3 (4.48%)	3 (3.41%)	17 (43.59%)	
**Lifetime social anxiety disorder**	39 (20.10%)	–	28 (31.82%)	11 (28.21%)	
**Lifetime generalized anxiety disorder**	41 (21.13%)	–	31 (35.23%)	10 (25.64%)	
**Lifetime posttraumatic stress disorder**	25 (12.89%)	–	14 (15.91%)	11 (28.21%)	
**Lifetime attention-deficit hyperactivity disorder**	23 (11.86%)	–	16 (18.18%)	7 (17.95%)	
**Lifetime substance use disorder**	4 (2.06%)	–	3 (3.41%)	1 (2.56%)	
**Smartphone type**					0.220
Android	35 (18.04%)	10 (14.93%)	17 (19.32%)	8 (20.51%)	
iOS	159 (81.96%)	57 (85.07%)	71 (80.68%)	31 (79.49%)	
**Site**					0.189
New York	104 (53.61%)	45 (67.16%)	30 (34.09%)	29 (74.36%)	
Chicago	90 (46.39%)	22 (32.84%)	58 (65.91%)	10 (25.64%)	

*MDD* major depressive disorder.

### Procedure and materials

All procedures were conducted in accordance with the Declaration of Helsinki and were approved by the Institutional Review Board of the New York State Psychiatric Institute. Written assent was obtained from adolescents under 18 years of age, with written consent provided by their parents or legal guardians; participants aged 18 years provided their own informed consent. At baseline, trained interviewers administered the DSM-5 version of the K-SADS-PL for lifetime psychiatric disorders, the WASI-II two-subtest form for verbal and nonverbal intelligence, and the CDRS-R for depressive severity. In a separate session, EEG data were recorded during a revised Chatroom Task. To obtain daily mood ratings and keyboard inputs over the 12-month follow-up, participants installed the Effortless Assessment Research System (EARS) app [39] on their personal smartphones and were instructed to keep the app running while using their phones as usual. Clinical interviews assessing depressive symptoms were re-administered at the 12-month follow-up. The present analyses were restricted to adolescents who completed both the baseline and 12-month clinical assessments.

### Clinical interviews

During the clinical assessment, trained study staff administered the K-SADS-PL to assess lifetime psychiatric disorders; interrater reliability was excellent (Cohen’s κ = 0.98). Depressive symptom severity was assessed at baseline and at the 12-month follow-up with the CDRS-R, a 17-item clinician-rated interview. Total CDRS-R scores range from 17–113, with higher scores reflecting more severe depressive symptoms; scores ≥55 are conventionally considered indicative of a current MDD episode. Anxiety symptoms were assessed at baseline and the 12-month follow-up using the Social Anxiety subscale of the Inventory of Depression and Anxiety Symptoms–II (IDAS-II; sum of items 15, 18, 20, 41, 47, and 99) [40], a 99-item self-report measure of internalizing psychopathology. Internal consistency was good (α = 0.78–0.81).

### Experimental task

Participants completed a revised Chatroom Task adapted from prior peer-evaluation paradigms [41]. The paradigm was administered across two sessions and involved deception. Participants were told that the study examined how adolescents interact online in collaboration with laboratories at other sites, and that following the EEG session they would be connected with another adolescent for approximately 10 min of online conversation. No such interaction took place.

At the first session, approximately one week before EEG acquisition, participants were photographed and created a brief profile of their interests (e.g., favorite foods, hobbies), which they were told would be uploaded to the study system and reviewed by other participants. Participants then viewed photographs and profiles of 100 purported peers (50 male, 50 female) and designated exactly 50 with whom they were interested in chatting (high-value) and 50 with whom they were not (low-value); selections could be revisited and revised before submission. Photographs were drawn from a teen face emotion stimulus set developed for use in adolescent peer-evaluation paradigms [41]; full stimulus details are provided in the Supplementary Materials.

During the EEG session, participants received acceptance or rejection feedback for each purported peer. Each trial began with a previously reviewed peer photograph presented for 2000 ms alongside a reminder of the participant’s prior selection (“interested” or “not interested”), followed by a 1000 ms fixation cross. Feedback was then presented for 2000 ms, with acceptance and rejection signaled by a green check mark or red “X,” respectively. Participants subsequently rated how the feedback made them feel on a visual analogue scale (0 = Very Good–100 = Very Bad) presented for 4000 ms, followed by a jittered intertrial interval of 1500–1700 ms. The task included 50 acceptance and 50 rejection trials, balanced across peer value (25 trials per cell), with trial order pseudorandomized such that no more than two consecutive trials of the same feedback type occurred.

The task yielded four within-subject conditions: acceptance and rejection from high-value and low-value peers. As the primary hypothesis concerned neural sensitivity to social rejection, primary analyses focused on feedback from high-value peers. Rejection by a desired peer was considered the condition most directly relevant to rejection sensitivity, as it more closely reflects the social experiences theorized to contribute to depressive vulnerability [42]. ΔP3 (rejection vs. acceptance) was accordingly computed from high-value peer trials. Analyses incorporating peer value as a within-subject factor are reported in Supplementary Table 2.

Following the task and before debriefing, participants completed a brief questionnaire that included ratings of task interest, affective responses to acceptance and rejection, and a yes/no item assessing whether they believed they would actually be chatting with one of the peers after the EEG session; participants who responded “no” were asked to provide an explanation. All participants were fully debriefed on the same day as the EEG session.

### Electrophysiological recordings and data reduction

EEG was recorded during the revised Chatroom task using a 32-channel ActiCHamp system (Brain Products, Munich, Germany) arranged according to the international 10–20 system and acquired with BrainVision Recorder. Signals were sampled at 500 Hz and referenced online to FCz; the ground electrode was placed between Fp1 and Fp2. Vertical and horizontal EOG were recorded, and all electrode impedances were kept below 20 kΩ. All tasks underwent the same preprocessing pipeline, implemented in MATLAB using custom scripts and the EEGLAB toolbox [43, 44]. Continuous EEG data were band-pass filtered between 1 and 40 Hz. The clean_rawdata routine was then used to identify and remove artifact-contaminated segments, including channels with more than 5 s of flat signal or correlations below 0.70 with neighboring channels [45]. Artifact Subspace Reconstruction was subsequently applied to attenuate brief, high-amplitude noise using a 500 ms sliding window advanced in 250 ms steps; components exceeding 20 standard deviations above the artifact-free reference were corrected [46]. Independent component analysis was then performed and components classified using ICLabel [47]; those identified as ocular, muscular, or other non-neural sources were removed before back-projection. These parameters were based on established recommendations and prior validation work for the respective routines rather than tuned to the present results.

Following automated preprocessing, all epochs were visually inspected and those containing residual artifact were excluded. Participants for whom more than 15% of trials were rejected on this basis were excluded from ERP analyses, ensuring an adequate number of artifact-free trials per participant while limiting the influence of residual artifact on ERP estimates.

### Event-Related potential (ERP) analysis

**Fig. 1:**
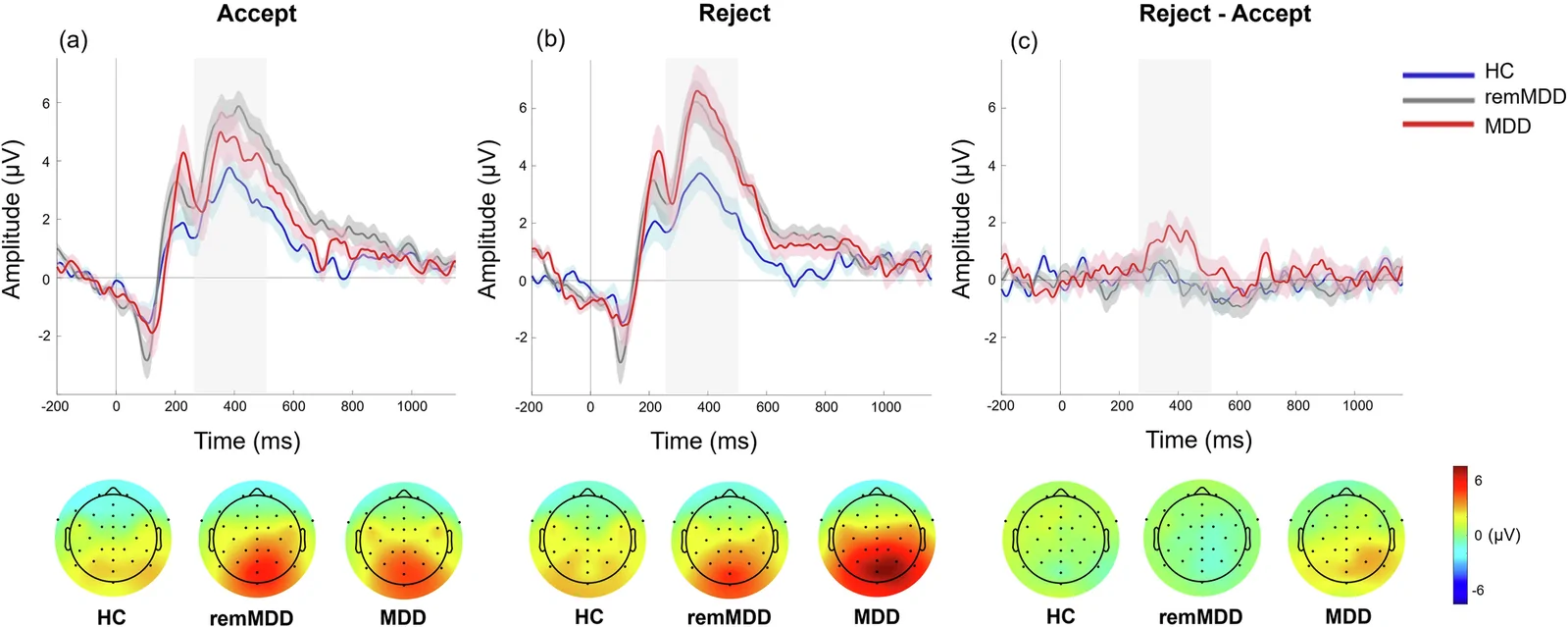
Group differences in EEG responses to peer acceptance and rejection. Feedback-locked event-related potentials (ERPs) at Pz for healthy controls (HC; blue), adolescents with remitted major depressive disorder (remitted MDD; gray), and adolescents with current MDD (red). **a** Grand-average waveforms to accept feedback. **b** Grand-average waveforms to rejection feedback. Time 0 ms marks feedback onset. **c** Rejection–acceptance difference waves (ΔP3 = rejection − acceptance) for each group. The shaded interval in panel (**c**) denotes the 250–500 ms time window used to quantify P3 amplitude, during which a significant main effect of the group was observed. Scalp topographies below each panel show mean amplitude in this window for each group, corresponding to the feedback condition depicted in the panel directly above.

Stimulus-locked ERPs were averaged separately for acceptance and rejection feedback. Epochs extended from −200–1200 ms relative to feedback onset ( − 200–0 ms pre-feedback baseline). Consistent with the maximal P3 across participants, scalp topographies, and prior work [8, 16], P3 amplitude was quantified at Pz as the mean voltage from 250–500 ms post-feedback. For longitudinal models, ΔP3 was calculated as the difference between rejection and acceptance conditions (hereafter referred to as ΔP3). As the task delivers valenced social feedback, we additionally examined whether the earlier feedback-locked activity showed characteristics of a reward positivity (RewP). Following convention in the social feedback literature [20, 48], RewP amplitude was quantified as the mean voltage from 250–350 ms post-feedback at Cz. Separate averages were computed for acceptance and rejection feedback within each diagnostic group (see Fig. 1 for grand-average waveforms at Pz and Supplementary Figure 1 for frontal and frontocentral sites).

### Daily mood

Daily mood was assessed via the question, “In general, how have you been feeling over the last day?”, which referred to the 24 h preceding delivery of the prompt. This question was sent via the EARS app at 12:00 p.m. each day and remained available until 11:59 p.m. Ratings were made on a sliding visual analogue scale ranging from 0 (very negative) to 100 (very positive). Although participants had 12 h to respond, most responses were completed relatively soon after prompt delivery (median response latency = 14.64 min; M = 77.86, SD = 145.67). Additional details on daily mood data processing are provided in the Supplementary Materials.

### EARS keyboard input preprocessing

Passive smartphone keyboard inputs from the participants’ phones only were collected using EARS [39]. Importantly, EARS recorded only participants’ self-generated (outgoing) keyboard input across all applications (e.g., text messaging, social media, web searches); no third-party inputs were captured and the intended recipient or communication partner could not be identified. All data were encrypted and stored on a secure cloud-based server accessible only to the study team; Ksana Health did not have access to participant data. Details on text data acquisition, linguistic preprocessing, manual coding reliability (e.g., inter-rater kappa), and additional privacy protections are provided in the Supplementary Materials.

### Detection of friendship language

A dataset of 10,000 random entries from 20 participants, selected to represent the diagnostic groups, study sites, sex, and age range of the sample, was labeled for friendship-related content by a trained coder. Entries were coded as friendship-related when the language described a friendship interaction or social situation, including conflict, validation, instrumental support, and companionship; communication addressed to a friend that did not describe a friendship interaction or social situation was not included (see Supplementary Materials for selection and coding details). The data were split into 70, 15, and 15% training, validation, and test sets, stratified by participant and label. We fine-tuned FLAN-T5-base, an open-source encoder–decoder model with 0.2 billion parameters [49]; see Supplementary Materials for fine-tuning details]. On the held-out test set, the model achieved an F1 score of 0.837. For each participant, friendship-focused communication was computed as the proportion of classified entries labeled friendship-related across the 12-month follow-up (M = 2.37%, SD = 1.57%, range = 0–8.79%, skewness = 1.76). The metric did not differ across diagnostic groups, (*p* = 0.324).

### Data analytic approach

Among the 194 enrolled adolescents, 15 were excluded due to missing EEG data (*n* = 9; 7 remitted MDD, 2 healthy controls) or poor EEG data quality (*n* = 6; 2 current MDD, 1 remitted MDD, 3 healthy controls), defined as excessive artifacts (e.g., muscle activity, eye movements, or electrode noise) and/or insufficient clean data after preprocessing that rendered ICA decomposition unreliable. The final sample comprised 179 adolescents: current MDD (*n* = 37), remitted MDD (*n* = 80), and healthy controls (*n* = 62). All analyses were conducted in R (version 4.3.1) using two-sided tests with an alpha level of 0.05. Continuous predictors were mean-centered, and ΔP3 and friendship-focused social communication were standardized (z-scored) to facilitate interpretation of regression coefficients.

Follow-up retention was high with no attrition bias (completers vs. noncompleters, all ps > 0.05), and split-half reliability of the P3 measures was good to acceptable across conditions and for the ΔP3; full details are provided in the Supplementary Materials.

#### Baseline group differences

Diagnostic-group differences in P3 amplitude were tested in a linear mixed model with Group (current MDD, remitted MDD, healthy control) and Feedback Type (acceptance, rejection) as fixed effects, their interaction, age, sex, and site as covariates, and a by-participant random intercept. Retaining feedback type as a within-subject factor allows the contribution of each condition to be evaluated directly. Significant interactions were decomposed with Tukey-corrected comparisons of feedback type within each group and of group within each feedback type. To summarize these condition-level effects in the single index used in the longitudinal models, we additionally modeled ΔP3 (P3 amplitude for rejection minus acceptance feedback at Pz) in a general linear model with Group as a between-subject factor and the same covariates, following significant omnibus effects with Tukey-corrected pairwise comparisons.

#### Prediction of depressive symptoms and daily mood

To test whether ΔP3 prospectively predicted clinician-rated depressive severity at follow-up, we fit negative binomial generalized linear models (GLMs) with 12-month CDRS-R score as the outcome. Predictors included Group, ΔP3, and their interaction (Grou*p* × ΔP3), with age, sex, site, and baseline CDRS-R entered as covariates. To verify that results did not depend on this operationalization, these models were repeated as a sensitivity analysis using a residualized rejection P3, computed by regressing the P3 to rejection on the P3 to acceptance (Supplementary Table 3). Generalized variance inflation factors (GVIF^(1/(2·df))) were all below 2.0, indicating acceptable collinearity. Significant interactions were probed using simple-slope analyses within each diagnostic group, estimating the association between ΔP3 and follow-up CDRS-R at ±1 SD of ΔP3. Regression diagnostics (residual plots, leverage, Cook’s distance) were inspected to confirm model assumptions; sensitivity analyses with robust estimators yielded similar results. To evaluate whether the prospective association was specific to depressive symptoms, the model was repeated with 12-month IDAS Social Anxiety as the outcome [40], adjusting for log-transformed baseline IDAS Social Anxiety scores and the same covariates (Supplementary Table 4).

We next examined whether baseline ΔP3 was associated with adolescents’ emotional experience in daily life. Average daily mood was regressed on ΔP3, controlling for age, sex, site, and Group. In secondary models, baseline CDRS-R was added as a covariate to test whether ΔP3 was associated with everyday mood beyond baseline depressive severity. As in the symptom models, we examined Grou*p* × ΔP3 interactions and probed significant effects with simple slopes. Exploratory analyses examining smartphone communication as a moderator are reported in Supplementary Materials.

## Results

### Baseline group differences

We first examined whether the early feedback-locked activity showed characteristics of a RewP. In the conventional RewP measurement window (250–350 ms), amplitudes at Fz, FCz, and Cz did not differ between acceptance and rejection feedback in any group (ps > 0.05), and the scalp distribution was posterior-maximal rather than frontocentral (Supplementary Figure 1). We therefore did not observe a RewP-like effect in these data. Condition-related differences instead emerged in the later centro-parietal positivity.

The Grou*p* × Feedback Type interaction was significant, F(2, 176) = 4.84, *p* = 0.009 (Fig. 1). The interaction was driven by current MDD: the Current MDD × Rejection term was significant (b = 1.43, SE = 0.46, t = 3.10, *p* = 0.002), whereas the Remitted MDD × Rejection term was not (b = 0.38, SE = 0.36, t = 1.07, *p* = 0.287). Within-group comparisons of rejection versus acceptance (Tukey-adjusted) indicated that only current MDD showed a significant valence difference, with larger P3 amplitudes to rejection than acceptance (*p* = 0.008); neither healthy controls (*p* = 0.128) nor remitted MDD (*p* = 0.889) differentiated the two conditions.

Between-group comparisons within each condition contextualize these findings. In the rejection condition, both current MDD (*p* = 0.001) and remitted MDD (*p* = 0.002) showed larger P3 amplitudes than healthy controls and did not differ from each other (*p* = 0.524). In the acceptance condition, only remitted MDD exceeded healthy controls (*p* = 0.011); current MDD did not differ significantly from controls (*p* = 0.132). The current MDD P3 amplitudes therefore reflected amplified responses to rejection rather than attenuated responses to acceptance, whereas remitted MDD showed a generalized, valence-nonspecific elevation (Supplementary Table 1). Including peer value as a within-subject factor did not alter this pattern. Group differences in P3 amplitude were confined to high-value peer trials, on which both remitted MDD (estimate=2.08, SE = 0.49, *p* < 0.001) and current MDD (estimate=2.17, SE = 0.65, *p* = 0.002) exceeded healthy controls; neither group differed from controls on low-value peer trials (ps > 0.94; Supplementary Table 2).

Adolescents with current MDD showed larger differentiation than both healthy controls (*p* = 0.004, d = 0.81) and remitted MDD (*p* = 0.043, d = 0.58), whereas remitted MDD and healthy controls did not differ (*p* = 0.463, d = 0.23). ΔP3 was positively correlated with baseline CDRS-R scores across the full sample (r = 0.19, *p* = 0.030). At debriefing, 83.6% of participants reported that they had believed they would be chatting with one of the peers following the EEG session. Believability did not differ significantly across diagnostic groups, χ²(2) = 0.59, *p* = 0.745.

### Prediction depressive symptoms and daily mood

The Grou*p* × ΔP3 interaction predicting 12-month depressive severity was significant, B = 0.092, 95% CI [0.002, 0.181], *p* = 0.045 (Table 2). Results were comparable when the residualized rejection P3 was substituted for ΔP3. The interaction with current MDD was of similar direction and magnitude (b = 0.17, SE = 0.09, *p* = 0.051), with no comparable effect in remitted MDD (b = −0.06, SE = 0.06, *p* = 0.269). As responses to the two feedback conditions were highly correlated (r = 0.83), the residualized measure has a restricted range and correspondingly less precise estimates (Supplementary Table 3). Simple-slope analyses indicated that greater ΔP3 predicted higher follow-up depressive symptoms only among adolescents with current MDD at baseline, whereas associations in the remitted MDD and healthy control groups were small and non-significant (Supplementary Figure 2).

**Table 2:** Baseline ΔP3 to peer feedback and diagnostic group predicting depressive symptom severity (CDRS-R) at the 12-month follow-up assessment.

Parameter	b	Std. Error	p value	95% CI (β)
(Intercept)	2.369	0.426	2.63e–08	[1.535, 3.204]
Age	−0.006	0.018	0.724	[−0.040, 0.028]
Site	−0.049	0.052	0.346	[−0.152, 0.053]
Sex	0.059	0.055	0.281	[−0.048, 0.166]
Baseline depressive symptoms	0.197	0.115	0.089	[−0.030, 0.423]
ΔP3	0.020	0.025	0.418	[−0.028, 0.068]
MDD (relative to HC reference)	0.060	0.131	0.648	[−0.197, 0.317]
remMDD (relative to HC reference)	0.078	0.058	0.180	[−0.036, 0.193]
ΔP3 x MDD	0.092	0.046	0.045	[0.002, 0.181]
ΔP3 x remMDD	−0.036	0.030	0.240	[−0.095, 0.024]

*HC* healthy controls, *MDD* adolescents with current major depressive disorder, *remMDD* adolescents with remitted major depressive disorder.

To assess the specificity of the prospective association with depressive symptoms, we repeated the model with 12-month IDAS Social Anxiety as the outcome. Baseline ΔP3 did not predict 12-month social anxiety symptoms (b = 0.01, SE = 0.09, *p* = 0.946), and this association did not differ by group (ΔP3 × current MDD: b = 0.05, SE = 0.15, *p* = 0.744; ΔP3 × remitted MDD: b = −0.02, SE = 0.11, *p* = 0.832). Baseline social anxiety significantly predicted follow-up social anxiety symptoms in the same model (b = 0.65, SE = 0.21, *p* = 0.002; Supplementary Table 4).

**Fig. 2:**
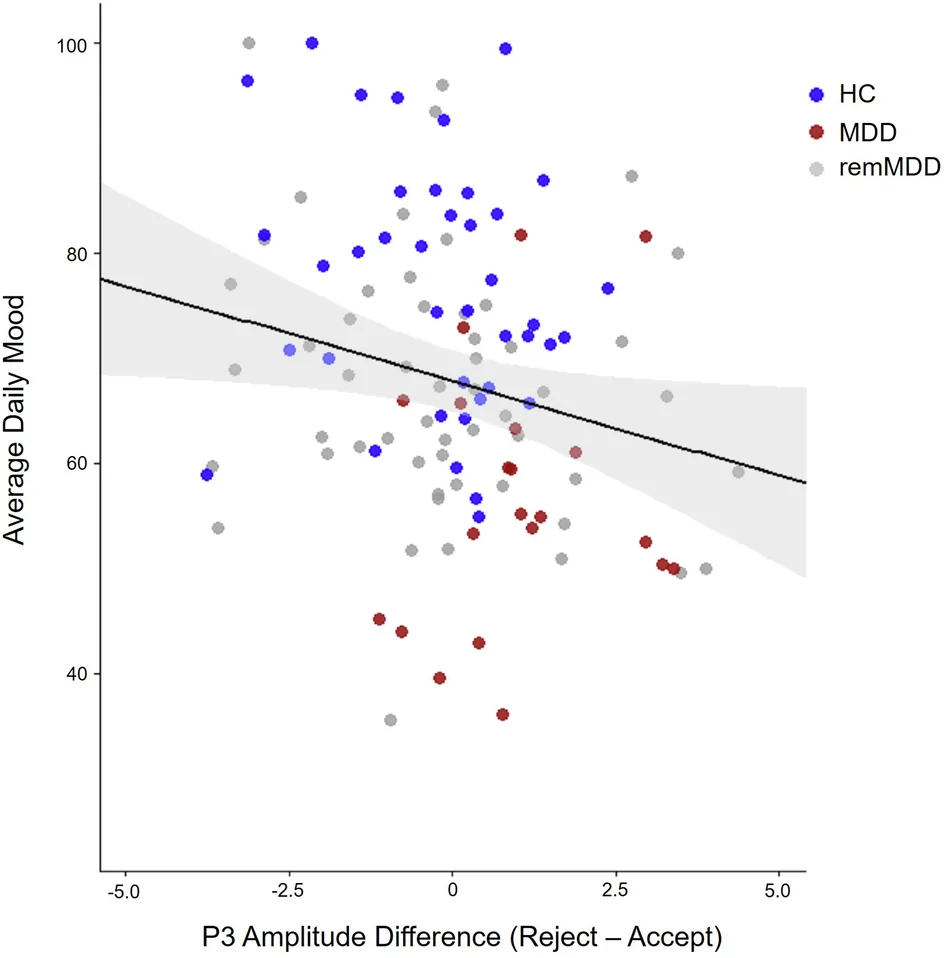
Association between neural sensitivity to peer rejection and average daily mood. Each point represents one participant, colored by the diagnostic group (HC healthy controls, MDD adolescents with current major depressive disorder, remMDD adolescents with remitted major depressive disorder). The x-axis shows ΔP3 (P3 amplitude to rejection minus acceptance feedback at Pz) measured at baseline. The y-axis shows average daily mood, computed as the mean of smartphone-based ecological momentary assessment ratings (0 = very negative, 100 = very positive) over the follow-up period. The solid line depicts the fitted regression line for the full sample, and the shaded band represents the 95% confidence interval from a model adjusting for age, sex, and site.

The Grou*p* × ΔP3 interaction was not significant for average daily mood (*p* = 0.334) and was therefore removed from the final model. Across the full sample, larger ΔP3 was associated with worse average daily mood over 12 months, b = −1.673, *p* = 0.045 (Fig. 2). This association was attenuated and no longer significant when baseline depressive severity was included as a covariate (b = −1.03, SE = 0.74, *p* = 0.169).

### Exploratory analyses focused on adolescent smartphone communication

The three-way Grou*p* × ΔP3 × Friendship interaction predicting 12-month depressive severity was significant for current MDD relative to healthy controls (b = −0.58, SE = 0.18, *p* = 0.001) but not for remitted MDD (*p* = 0.572; Table 3). Full model results, simple-slope estimates, and the corresponding figure are reported in the Supplementary Materials (Supplementary Figure 3). Johnson–Neyman analysis indicated that, within the current MDD group, the association between ΔP3 and 12-month depressive severity was significantly positive at friendship-focused communication values below z = 0.12 and significantly negative above z = 0.99, with no significant association in the intervening range (Supplementary Figure 4). Conditional slopes were non-significant throughout the observed range of the moderator in the healthy control and remitted MDD groups.

**Table 3:** Predicting 12-month depressive symptom severity (CDRS-R) from baseline ΔP3 to peer feedback, friendship-language frequency, diagnostic group, and their interactions.

Predictor	Estimate (b)	Std. Error	p value
Intercept	2.587	0.522	< 0.001
Age	−0.032	0.022	0.131
Site	0.028	0.062	0.655
Sex	0.094	0.070	0.178
CDRS-R at baseline	0.230	0.143	0.109
MDD group (relative to HC reference)	0.238	0.154	0.124
remMDD group (relative to HC reference)	0.084	0.074	0.258
ΔP3 (z)	0.028	0.074	0.704
Friendship-related text entries	0.064	0.052	0.215
MDD × ΔP3	0.205	0.112	0.067
remMDD × ΔP3	−0.070	0.085	0.411
MDD × Friendship-related text entries	0.041	0.119	0.732
remMDD × Friendship-related text entries	−0.049	0.077	0.526
ΔP3 × Friendship-related text entries	−0.005	0.086	0.951
MDD × ΔP3 × Friendship-related text entries	−0.578	0.181	0.001
remMDD × ΔP3 × Friendship-related text entries	0.055	0.097	0.572

*HC* healthy controls, *MDD* adolescents with current major depressive disorder, *remMDD* adolescents with remitted major depressive disorder.

## Discussion

The present study examined the prospective links between neural sensitivity to peer-evaluative feedback and the longitudinal course of adolescent depression, integrating EEG-based measures with smartphone-derived digital phenotyping. Compared to both healthy controls and adolescents with remitted MDD, adolescents with current MDD exhibited markedly greater P3 to peer rejection relative to acceptance (ΔP3). Although ΔP3 is a difference score, a condition-level analysis confirmed that the group difference was driven primarily by amplified P3 responses to rejection rather than attenuated responses to acceptance; indeed, P3 amplitudes to acceptance feedback were comparable or larger in clinical groups relative to healthy controls (Fig. 1a).

Within the current MDD group, higher baseline ΔP3 prospectively predicted greater depressive severity 12 months later after accounting for baseline depressive symptoms. Exploratory analyses of text-message content further indicated that the association between neural sensitivity to peer rejection and symptom persistence depended on real-world social behavior, with higher levels of friendship-focused smartphone communication attenuating the link between ΔP3 and later depressive severity.

The pattern of ΔP3 partially supported the *a priori* hypothesis of a graded effect across groups. ΔP3 was largest in adolescents with current MDD, whereas remitted youth did not differ from healthy controls. This profile suggests that heightened neural sensitivity to peer rejection, at least as indexed by the P3 amplitude difference, is more tightly linked to acute depressive state than to a scar of depression or a persistent trait-like liability [6, 7, 50]. Adolescents with current MDD appear to show an exaggerated differentiation between rejection and acceptance feedback, consistent with preferential allocation of attentional and motivational resources to socially threatening cues [8, 16, 51]. Once symptoms remit, however, P3 responses to peer feedback may return to a more normative range, even among youth who remain at elevated risk for recurrence. This state-dependent profile underscores the possibility that social–affective neural responses in adolescence are modifiable targets rather than fixed attributes [34].

Beyond its association with concurrent severity, ΔP3 may be a predictive marker of a more persistent depressive course, consistent with other longitudinal studies identifying neural prognostic markers [22, 29, 52]. This pattern aligns with interpersonal and stress-generation models of depression [53], which propose that heightened sensitivity to social threat may foster maladaptive responses such as rumination, excessive reassurance-seeking, and social withdrawal that maintain depressive states [50, 54, 55]. Identifying a neurophysiological marker that tracks symptom persistence raises the possibility that ΔP3 could be incorporated into risk stratification efforts [20, 51, 56] helping to delineate a subgroup of adolescents with high rejection sensitivity who may be especially likely to benefit from interventions targeting social-cognitive biases and interpersonal coping [16, 34, 57].

The association between ΔP3 and average daily mood aligns with experience sampling research showing that adolescents’ typical mood levels and affective dynamics are closely tied to depressive symptoms and risk [25–27]. It is also consistent with interpersonal models of depression, which propose that heightened neurocognitive sensitivity to negative social cues can bias the interpretation of daily interactions, reinforcing low mood and setting the stage for withdrawal and ongoing distress. Notably, the association between ΔP3 and daily mood was observed across the full sample regardless of diagnostic status, suggesting that neural sensitivity to peer rejection is a transdiagnostic risk factor for affective disruption in adolescence [34, 53].

Building on prior work from our group demonstrating that adolescents’ smartphone social communication covaries with daily mood [24], these exploratory analyses offered a complementary, real-world lens on how ΔP3 may translate into everyday social behavior and subsequent symptom burden. Among adolescents with current MDD, elevated ΔP3 was most strongly associated with persistent depressive symptoms at follow-up in those who engaged in relatively low levels of friendship-focused smartphone communication—operationalized here as the proportion of all keyboard input whose content pertains to friendship themes (e.g., companionship, social activities, interpersonal conflict), irrespective of the recipient or application, rather than a measure of social network size or frequency of contact with specific friends. As such, it remains possible that unmeasured aspects of social connection contributed to the observed associations. This pattern is consistent with developmental models of social withdrawal and depression, in which reduced engagement with peers limits opportunities for positive social reinforcement and corrective interpersonal experiences, allowing negative social schemas to consolidate [58–60]. In contrast, as current MDD adolescents who engaged in high levels of friendship-focused smartphone communication exhibited *decreases* in depressive severity over time, maintaining contact with friends, even through low-effort channels such as texting, may provide a form of social scaffolding that dampens the impact of rejection sensitivity on longer-term mood [61–63]. Although these findings are preliminary and based on relatively coarse linguistic categories, they highlight a promising pathway linking neural and digital markers that warrants more fine-grained testing in future work, including studies that directly evaluate whether supporting peer engagement can mitigate risk among adolescents with elevated neural rejection sensitivity [64, 65].

Several limitations should be noted. First, the sample was predominantly female, which is consistent with known sex differences in adolescent depression but limits power to examine sex differences and may constrain generalizability. Second, all digital data were derived from participants who owned compatible smartphones and agreed to install the EARS keyboard, so the linguistic analyses may under-represent adolescents with lower digital engagement or greater privacy concerns. Third, the observational design permits inferences about prospective associations but not definitive causal conclusions; unmeasured factors such as family environment, treatment exposure, or broader stress contexts may contribute to the observed links. Fourth, the current MDD subgroup was relatively small (*n* = 37), and key prospective findings were primarily driven by this group; replication in larger samples is essential. Last, EEG data were collected at a single time point, so within-person changes in ΔP3 from the depressed to remitted state could not be evaluated. Longitudinal designs with repeated EEG assessments will be important to determine whether interventions targeting rejection sensitivity also modify this neural signal.

In summary, this study identifies a neural signature of peer rejection sensitivity, the ΔP3, that is elevated during acute adolescent depression and prospectively associated with both clinician-rated depressive severity and average daily mood. The impact of this neural risk marker appears to depend on adolescents’ ongoing patterns of social engagement, with sustained friendship-focused texting attenuating risk. These findings underscore the value of integrating neural measures with digital indicators of real-world behavior to refine risk prediction and inform personalized, developmentally sensitive interventions for depressed adolescents.

## Supplementary information

Supplemental material

## Data Availability

Data are publicly available through the National Data Archive.
