# Supplementary material for: Neural sensitivity to peer rejection predicts depressive symptoms and daily mood in adolescents
Source: NPP Digit Psychiatry Neurosci. 2026 Oct 22;4:21. doi: 10.1038/s44277-026-00073-0 (PMC13691077; doi:10.1038/s44277-026-00073-0)
Supplement: Supplementary file 1 [file 44277_2026_73_MOESM1_ESM.docx]

**Supplementary Materials**

**Methods**

**Experimental Task**

**Peer Stimuli**. Photographs were drawn from a teen face emotion stimulus set developed for use in adolescent peer-evaluation paradigms [[1]](https://www.zotero.org/google-docs/?JBxsXT), comprising digital headshots of 11- to 17-year-old actors of varied ethnicities posing happy expressions with direct gaze. As described by Guyer and colleagues, the photograph most clearly conveying happiness was selected for each actor, and attractiveness was not controlled in order to maintain a stimulus set reflecting the range of peers typically encountered by adolescents. Profiles describing interests and hobbies were constructed by the study team to be age-appropriate and plausible.

**Daily Mood**

For each participant, we removed duplicate responses on the same day and any values outside the allowable range. We then computed average daily mood as the mean of all valid ratings across the 12-month follow-up. The response rate was 52.7%. A threshold of fewer than 10 valid mood ratings over 12 months was predefined as “extremely sparse” data for exclusion from average daily mood analyses; however, no participants met this criterion and accordingly, adolescents contributed all available mood observations. Higher scores indicate a more positive overall affective climate across the follow-up period.

**Data analytic approach**

Baseline-to-follow-up intervals averaged 12.23 months (*SD* = 0.88), with 89.6% of participants completing the 12-month assessment. Longitudinal models were accordingly limited to follow-up completers. To examine attrition bias, completers and noncompleters at 12 months were compared on baseline demographic, clinical, and neurobiological measures. No variable differentiated the two groups (all *p*s > .05).

Split-half reliability was estimated by correlating P3 amplitudes derived from odd- versus even-numbered trials, corrected with the Spearman–Brown prophecy formula. Reliability was good for the condition-level amplitudes (acceptance P3, *r* = .72; rejection P3, *r* = .81) and acceptable for the difference score (ΔP3, *r* = .71), consistent with values reported for comparable peer-feedback P3 paradigms [[2]](https://www.zotero.org/google-docs/?EKU0Fe).

**Data Privacy Protections**

Data collected by the Effortless Assessment Research System (EARS) app [[3]](https://www.zotero.org/google-docs/?d9stVp) were encrypted and stored on a cloud-based server. Only the study team had access to the data; other parties involved in data collection and storage, such as Ksana Health, did not have access. Data collection involved a logging tool that recorded participants' self-generated keyboard inputs only; no third-party keyboard inputs were recorded. Detailed information on privacy protections can be found in [[3]](https://www.zotero.org/google-docs/?ESyYt9) and on the Ksana Health privacy policy page.

Deidentified data were submitted to the National Data Archive (NDA) in accordance with the National Institute of Mental Health's Data Sharing Policy. To further safeguard privacy, all shared data were reviewed to exclude content that could compromise third-party privacy. To protect participant confidentiality, raw key input data, which can include potentially identifying information (e.g., names, social media handles, uncommon word strings), were not submitted to the NDA. Only daily aggregates of key input data (e.g., daily word count) were submitted.

**Keyboard Input Preprocessing**

Each keyboard input (all characters typed in any app) was timestamped and labeled with the app used, then inputs were transformed into text entries (i.e., a message or search) using an algorithm identifying logical divisions (e.g., pauses, changes in app). Any blank entries, system-generated text, entries with only a single character (other than emojis), URLs, and duplicate entries within the same app with timestamps separated by less than 1s were discarded. Text data underwent a multi-layered de-identification process to protect participant privacy. Spell-correction and language detection was performed using a HIPAA-compliant instance of GPT3.5 for the entries used for model validation. Entries longer than 3 words [[4]](https://www.zotero.org/google-docs/?fzrDKL) determined by GPT3.5 to be non-English were translated using a HIPAA-compliant instance of GPT4. Fewer than 1% of text entries were identified as another language (Supplementary Materials). Models were deployed across all participants on all text entries with ≥ 3 words and more than 100 entries. This first criteria led to a sizable portion of entries being lost (remaining 60.6%, n=2.8 million entries), but no participants.

**Text Entry Deidentification**

First, custom in-house algorithms by the Ksana Health team removed sensitive personal information such as passwords, credit card numbers, and other structured sensitive data formats. Following this initial pass, we used Microsoft Presidio, an open-source, automated de-identification tool, to perform an additional layer of entity recognition and removal [[5]](https://www.zotero.org/google-docs/?bvPbZy). Presidio's algorithms were configured to detect and redact personally identifiable information (PII), including names, geographic locations, phone numbers, email addresses, and other common identifiers.

**Validation Dataset Curation**

We curated a dataset based on all participants in the current study with more than 500 entries and all entries ≥ 3 words (preliminary work indicated worse human and model performance for 1 and 2 word entries). Twenty participants were chosen, representing all three diagnostic categories, both study sites, and a range of sexes and ages. Each participant contributed 500 text entries, resulting in a dataset of 10,000. Thus, the dataset did not represent the most frequent communicators only, but instead adolescents who engaged in a wide range of communication. Before sampling the 500 entries from each participant, we categorized each entry into four length categories, and we used stratified sampling to select a representative distribution of entries from each category based on average percentages from the full dataset.

| **Entry Length** | **Criteria** | **Percentage Of Split** |
| --- | --- | --- |
| Short | 3<words<8 | 69% |
| Medium | 8<words<20 | 23% |
| Long | 20<words<50 | 7% |
| Very Long | >50 words | 1% |

Finally, this data was randomly shuffled across all 10000 entries to avoid any obvious relationships between the entries that could bias human coding.

**Validation Set**

A dataset of 10,000 random entries was selected from 20 participants carefully curated to represent across diagnostic categories, study sites, sex and ages A trained coder labeled each entry as friendship-related or not based on the following coding criteria: ‘about friendship interactions (could include at school), social situations like parties’. Interactions may include conflict, validation, instrumental support, companionship. Notably, this does not encompass any conversation with a friend, rather it refers specifically to language describing a friendship. Language explicitly discussing friendship-related activities/behaviors such as “let’s hang out” or “let’s link up” was coded as friendship-related, whereas vague references such as “let’s meet at such-and-such a location” were not. Affiliative words such as “girl” or “bro” were classified as friendship-related. The dataset was split into a 70-15-15 training, validation, and test sets. The data were stratified based on participant and label distributions. Training and validation sets were used throughout fine-tuning, whereas the test set was held out for evaluation [[6]](https://www.zotero.org/google-docs/?2sZj8W).

**Model performance**

We used an open-source encoder-decoder (text-text) language model, flan-T5-base, which has 0.2B parameters [[7]](https://www.zotero.org/google-docs/?a3FBlA). In the test dataset, relative to manual coding, precision of the model was 0.846, the recall was 0.828, and the F1 was 0.837. This was considered suitable for full-scale deployment on all text entries.

**Fine-tuning Details**

We fine-tuned a T5-base encoder–decoder model using parameter-efficient Low-Rank Adaptation (LoRA; [[8]](https://www.zotero.org/google-docs/?JiYQx6)) implemented with the Hugging Face *peft* library, while loading the frozen base model in 8-bit precision via *bitsandbytes* to reduce memory usage. LoRA adapters (rank = 16, α = 32, dropout = 0.05) were inserted into the query and value projection matrices of the attention layers, allowing only a small subset of parameters to be updated while keeping the original model weights fixed. Text messages were framed as instruction-style prompts and tokenized with percentile-based truncation lengths derived from the training corpus; labels were tokenized as target text and padding tokens were masked from the loss. Models were trained for 10 epochs using the Seq2SeqTrainer with a learning rate of 3×10⁻⁴, weight decay of 0.01, 100 warm-up steps, automatic batch sizing, and model selection based on validation F1 score.

**Exploratory analyses with smartphone communication as moderator of course**

To relate neural sensitivity to peer feedback to real-world social communication, smartphone communication collected over the 12-month follow-up. For each participant, we calculated the proportion of friendship-focused texts (friendship-focused text entries / total text entries) across the 12-month follow-up period, with higher values reflecting greater peer-oriented communication. We then tested whether friendship-focused smartphone language moderated the prospective association between ΔP3 and depressive severity. As follow-up CDRS-R scores were positively skewed and overdispersed, we used negative binomial GLMs with a log link. The primary model included Group, ΔP3, friendship-focused language, all lower-order interactions, and the three-way Group × ΔP3 × friendship-texting interaction, adjusting for baseline CDRS-R, age, sex, and site. Significant interactions were interpreted using simple-slope estimates at low (−1 SD), mean, and high (+1 SD) levels of friendship-focused texting within each diagnostic group. Coefficients from negative binomial models were exponentiated and reported as incidence-rate ratios (IRRs).

**Results**

**Exploratory Analyses Focused on Adolescent Smartphone Communication**

Exploratory analyses tested whether friendship-focused smartphone communication moderated the association between baseline ΔP3 and 12-month depressive severity. Depressive severity at follow-up (CDRS-R) was modeled using a negative binomial regression with baseline severity, ΔP3, friendship-focused communication (proportion of smartphone communication containing friend-related language), Group, and all two- and three-way interactions among Group, ΔP3, and friendship-focused communication, adjusting for age, sex, and site.

The three-way Group × ΔP3 × Friendship interaction was significant for current MDD relative to healthy controls (b = −0.58, SE = 0.18, p = .001) but not for remitted MDD (b = 0.06, SE = 0.10, p = .572). The effect was decomposed by examining the two-way ΔP3 × Friendship interaction within each group; it was significant only in current MDD. Simple-slope analyses indicated that among adolescents with current MDD reporting low friendship-focused communication (≈1 SD below the sample mean), a 1 SD increase in ΔP3 was associated with an approximately 2.3-fold increase in expected 12-month depressive severity, controlling for baseline severity and covariates. At mean levels the association was more modest, and at high levels (≈1 SD above the mean) it was attenuated and no longer clearly positive (Supplementary Figure S2). No comparable moderation was observed in remitted MDD or healthy controls.

**Supplementary Figures**

**Supplementary Figure 1.** Frontal and frontocentral responses to peer acceptance and rejection. Feedback-locked event-related potentials at Fz (top row) and Cz (middle row) for healthy controls (HC; blue), adolescents with remitted major depressive disorder (remitted MDD; gray), and adolescents with current MDD (red). (a) Grand-average waveforms to acceptance feedback. (b) Grand-average waveforms to rejection feedback. Time 0 ms marks feedback onset. (c) Rejection-minus-acceptance difference waveforms for each group. Shaded bands indicate the standard error of the mean, and the shaded interval marks the 250–350 ms window conventionally used to quantify the reward positivity (RewP). Scalp topographies below each column show mean amplitude in this window for each group, corresponding to the feedback condition depicted in the panels directly above.

**Supplementary Figure 2.** Simple slopes of ΔP3 (acceptance – rejection) predicting 12-month CDRS-R raw scores by diagnostic group. Predicted values are derived from a negative binomial generalized linear model (log link) adjusting for age, sex, site, and log-transformed baseline CDRS-R scores. Shaded bands represent 95% confidence intervals. The slope of ΔP3 was significant only in the current MDD group (*b* = 0.11, *p* = .045), indicating that greater neural sensitivity to peer rejection relative to acceptance at baseline predicted higher depressive symptom severity at follow-up. Slopes for the remitted MDD and healthy control groups were not significant (*ps* > .24). HC = healthy control; MDD = major depressive disorder; remMDD = remitted major depressive disorder.

**Supplementary Figure 3.** Simple slopes of ΔP3 (Rejection-Acceptance) predicting 12-month CDRS-R scores as a function of diagnostic group and friendship-focused smartphone communication. Predicted values are derived from a negative binomial generalized linear model (log link) adjusting for age, sex, site, and log-transformed baseline CDRS-R scores. ΔP3 and friendship-focused smartphone communication were z-standardized prior to model entry. Lines represent predicted CDRS-R scores at low (−1 SD; red), mean (orange), and high (+1 SD; blue) levels of friendship-focused smartphone communication, with shaded bands indicating 95% confidence intervals. MDD = major depressive disorder.

**Supplementary Figure 4.** Johnson-Neyman plot of the conditional association between ΔP3 (rejection − acceptance) and 12-month CDRS-R scores across levels of friendship-focused smartphone communication, by diagnostic group. Conditional slopes were derived from a negative binomial generalized linear model (log link) adjusting for age, sex, site, and log-transformed baseline CDRS-R scores. ΔP3 and friendship-focused smartphone communication were z-standardized prior to model entry. Shaded bands indicate 95% confidence intervals; regions in which the conditional slope differed significantly from zero (p < .05) are shown in blue and non-significant regions in pink, with dotted vertical lines marking the transition points. The horizontal black segment at zero indicates the observed range of friendship-focused communication within each group. MDD = major depressive disorder.

**Supplementary Table 1. Condition-Level Linear Mixed Model Predicting P3 Amplitude From Group, Feedback Type, and Their Interaction**

| **Predictor** | ***b*** | ***SE*** | ***t*** | ***p*** |
| --- | --- | --- | --- | --- |
| Intercept | 5.33 | 3.07 | 1.74 | .085 |
| Group: remitted MDD (vs. HC) | 1.81 | 0.62 | 2.92 | .004 |
| Group: current MDD (vs. HC) | 1.59 | 0.82 | 1.94 | .053 |
| Feedback: rejection (vs. acceptance) | −0.42 | 0.27 | −1.53 | .128 |
| Age | −0.25 | 0.19 | −1.29 | .198 |
| Sex | 1.08 | 0.59 | 1.84 | .068 |
| Site | 0.80 | 0.52 | 1.53 | .128 |
| Remitted MDD × Rejection | 0.38 | 0.36 | 1.07 | .287 |
| Current MDD × Rejection | 1.43 | 0.46 | 3.10 | .002 |

*Note*. Estimates are from a linear mixed model with a by-participant random intercept; degrees of freedom were approximated using the Kenward-Roger method. Reference levels: healthy controls (group) and acceptance (feedback type); positive coefficients therefore indicate greater P3 amplitude relative to these reference conditions. *b* = unstandardized coefficient; HC: healthy controls; MDD: adolescents with current major depressive disorder; remMDD: adolescents with remitted major depressive disorder; *SE* = standard error.

**Supplementary Table 2. Group Differences in P3 Amplitude by Peer Value**

| **Peer value** | ***Contrast*** | ***Estimate*** | ***SE*** | ***t*** | ***p*** |
| --- | --- | --- | --- | --- | --- |
| High | Remitted MDD − HC | 2.08 | 0.49 | 4.23 | < .001 |
| High | Current MDD − HC | 2.17 | 0.65 | 3.37 | .002 |
| Low | Remitted MDD − HC | 0.12 | 0.49 | 0.25 | .944 |
| Low | Current MDD − HC | −0.04 | 0.65 | −0.07 | .994 |

*Note*. Estimated marginal means from a linear mixed model including Group, Feedback Type, Peer Value, and their interactions, adjusting for age, sex, and site, with a by-participant random intercept. HC: healthy controls; MDD: adolescents with current major depressive disorder; remMDD: adolescents with remitted major depressive disorder; SE = standard error.

**Supplementary Table 3.** Baseline ΔP3 to peer feedback and diagnostic group predicting depressive symptom severity (CDRS-R) at the 12-month follow-up assessment

| **Parameter** | ***b*** | ***SE*** | ***z*** | ***p-value*** |
| --- | --- | --- | --- | --- |
| (Intercept) | 2.43 | 0.41 | 5.91 | < .001 |
| Age | −0.01 | 0.02 | −0.31 | .759 |
| Site | −0.05 | 0.05 | −0.95 | .340 |
| Sex | 0.06 | 0.05 | 1.03 | .304 |
| Baseline depressive symptoms | 0.18 | 0.11 | 1.59 | .112 |
| MDD (relative to HC reference) | 0.06 | 0.13 | 0.47 | .637 |
| remMDD (relative to HC reference) | 0.08 | 0.06 | 1.33 | .182 |
| Residualized rejection P3 | 0.04 | 0.05 | 0.76 | .446 |
| Residualized rejection P3 x MDD | 0.17 | 0.09 | 1.95 | .051 |
| Residualized rejection P3 x remMDD | −0.06 | 0.06 | −1.11 | .269 |

HC: healthy controls; MDD: adolescents with current major depressive disorder; remMDD: adolescents with remitted major depressive disorder; SE = standard error

**Supplementary Table 4.** Predicting 12-Month IDAS Social Anxiety From Baseline ΔP3, Group, and Their Interaction

| **Predictor** | ***b*** | ***SE*** | ***z*** | ***p-value*** |
| --- | --- | --- | --- | --- |
| Intercept | 0.03 | 0.95 | 0.03 | .973 |
| Age | −0.01 | 0.06 | −0.13 | .899 |
| Site | 0.01 | 0.17 | 0.04 | .972 |
| Sex | 0.09 | 0.18 | 0.48 | .629 |
| Baseline Social Anxiety | 0.65 | 0.21 | 3.07 | .002 |
| ΔP3 | 0.01 | 0.09 | 0.07 | .946 |
| MDD (relative to HC reference) | 0.16 | 0.30 | 0.55 | .584 |
| remMDD (relative to HC reference) | 0.16 | 0.22 | 0.76 | .448 |
| ΔP3 × MDD | 0.05 | 0.15 | 0.33 | .744 |
| ΔP3 × remMDD | −0.02 | 0.11 | −0.21 | .832 |

*Note*. Estimates are from a negative binomial generalized linear model with a log link. Reference levels: healthy controls (group). Baseline IDAS Social Anxiety scores were log-transformed. b = unstandardized coefficient; SE = standard error

**References**

[1. Guyer AE, McClure-Tone EB, Shiffrin ND, Pine DS, Nelson EE. Probing the Neural Correlates of Anticipated Peer Evaluation in Adolescence. Child Dev. 2009;80:1000–1015.](https://www.zotero.org/google-docs/?margCx)

[2. Pegg S, Lytle MN, Arfer KB, Kujawa A. The time course of reactivity to social acceptance and rejection feedback: An examination of event‐related potentials and behavioral measures in a peer interaction task. Psychophysiology. 2022;59:e14007.](https://www.zotero.org/google-docs/?margCx)

[3. Lind MN, Kahn LE, Crowley R, Reed W, Wicks G, Allen NB. Reintroducing the Effortless Assessment Research System (EARS). JMIR Ment Health. 2023;10:e38920.](https://www.zotero.org/google-docs/?margCx)

[4. Steinbrenner T, Lalk C, Targan K, Schaffrath J, Eberhardt S, Haberkamp A, et al. Explaining anxiety prediction in psychotherapy transcripts: The role of patient linguistic features and theoretical constructs. Behav Res Ther. 2025;194:104857.](https://www.zotero.org/google-docs/?margCx)

[5. PATCHIPALA SG. Data anonymization in AI and ML engineering: Balancing privacy and model performance using Presidio. 2023. 2023.](https://www.zotero.org/google-docs/?margCx)

[6. Treves IN, Bloom PA, Salem S, Durham K, Zaccaria V, Spence J, et al. In their own words: case studies of adolescent smartphone language preceding suicide-related hospitalizations. NPP—Digital Psychiatry Neurosci. 2026;4:5.](https://www.zotero.org/google-docs/?margCx)

[7. Chung HW, Hou L, Longpre S, Zoph B, Tay Y, Fedus W, et al. Scaling Instruction-Finetuned Language Models. J Mach Learn Res. 2024;25:1–53.](https://www.zotero.org/google-docs/?margCx)

[8. Hu EJ, Shen Y, Wallis P, Allen-Zhu Z, Li Y, Wang S, et al. LoRA: Low-Rank Adaptation of Large Language Models. 2021.](https://www.zotero.org/google-docs/?margCx)
